# Goose Astrovirus in China: A Comprehensive Review

**DOI:** 10.3390/v14081759

**Published:** 2022-08-12

**Authors:** Qinghe Zhu, Dongbo Sun

**Affiliations:** College of Animal Science and Veterinary Medicine, Heilongjiang Bayi Agricultural University, No. 5 Xinfeng Road, Sartu District, Daqing 163319, China

**Keywords:** goose astrovirus, epidemiology, genetic evolution, pathogenesis, cross-species transmission

## Abstract

Goose astroviruses (GoAstVs) are small non-enveloped viruses with a genome consisting of a single-stranded positive-sense RNA molecule. A novel GoAstV was identified in Shandong in 2016 and quickly spread to other provinces in China, causing gout in goslings, with a mortality rate of approximately 50%. GoAstV can also cause gout in chickens and ducks, indicating its ability to cross the species barrier. GoAstV has only been reported in China, where it has caused serious losses to the goose-breeding industry. However, in view of its cross-species transmission ability and pathogenicity in chickens and ducks, GoAstV should be a concern to poultry breeding globally. As an emerging virus, there are few research reports concerning GoAstV. This review summarizes the current state of knowledge about GoAstV, including the epidemiology, evolution analysis, detection methods, pathogenicity, pathogenesis, and potential for cross-species transmission. We also discuss future outlooks and provide recommendations. This review can serve as a valuable reference for further research on GoAstV.

## 1. Introduction

Based on the ability to infect mammalian and avian species, astroviridae can be divided into two genera: *Mamastrovirus* (MAstV) and *Avastrovirus* (AAstV) [1]. However, this classification based on the host taxa seems to be more complex, with astroviruses isolated from mammals and reptiles also clustering in the AAstV genera [2]. In addition, recently discovered novel astroviruses and genetic and evolutionary studies of astroviruses show that they have the potential to cross species barriers and adapt to new host species [3]. AAstV mainly infects turkeys, chickens, and ducks and can cause different diseases in different hosts, including hepatitis enteritis and kidney disease [1,4,5]. Goose astrovirus (GoAstV) is a newly discovered astrovirus, first identified in China in 2017. The virus causes gout and death in 4–16-day-old goslings [6,7,8]. Recent studies have reported that the infection and mortality rates of GoAstV in goslings and ducklings can reach 80% and 50%, respectively. GoAstV infections have resulted in serious economic losses to the poultry-breeding industry in the Chinese provinces of Jiangsu, Anhui, Shandong, Guangdong, Sichuan, and Liaoning [9,10,11]. As an emerging virus, research on GoAstV is still in the primary stage and many questions remain unresolved. The aim of this review was to describe the genome structure, classification, detection methods, epidemiology, gene evolution, and pathogenic characteristics of GoAstV.

## 2. Discovery and Epidemiology

**Figure 1:**
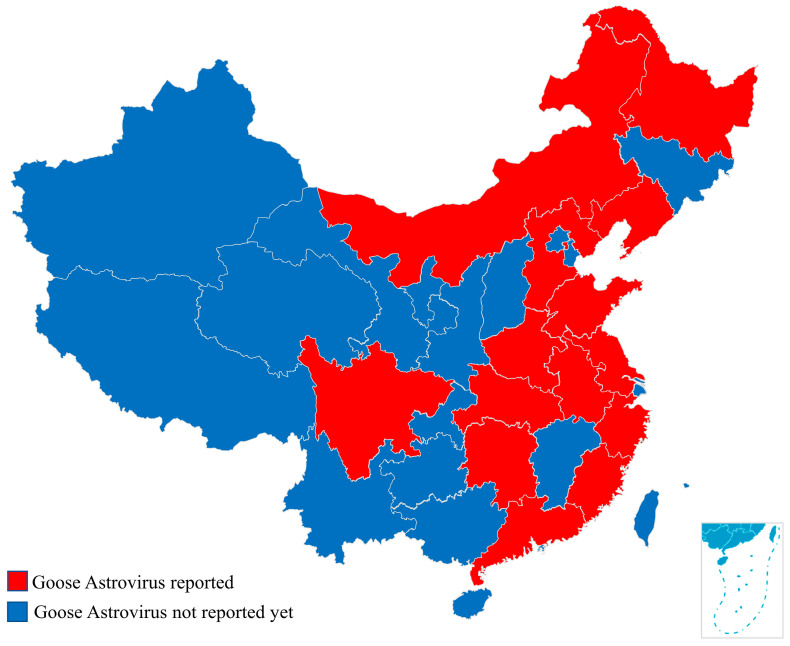
Provinces reporting GoAstV infections between 2014 and 2019.

A severe infectious disease characterized by gout, hemorrhage, and swelling of the kidneys has affected goslings in major goose-producing regions in China since November 2016. Zhang et al. (2018) reported a fatal infection of goslings characterized by visceral urate deposition and isolated a novel astrovirus (GoAstV) from dead goslings that was designated AAstV/Goose/CHN/2017/SD01. A similar disease was reproduced by experimental infection of healthy goslings, which fulfilled Koch’s postulates and provided the first demonstration of the etiological role of a genetically distinct astrovirus in the fatal infection of goslings [11]. In addition, Wang et al. (2021) revealed that strain TZ03 was also one of the causative agents of the ongoing goose gout disease in China [12]. However, phylogenetic tree analysis showed that TZ03 and AAstV/Goose/CHN/2017/SD01 strains belonged to different clusters. Researchers divided GoAstV into two genotypes: GoAstV-1 (G1) and GoAstV-2 (G2) [12]. Both genotypes cause the clinical symptoms of goose gout. Positive test results for GoAstV infection have been reported in Jiangsu, Shandong, Hubei, Anhui, Guangdong, Henan, Hebei, Liaoning, Hunan, Fujian, Zhejiang, Sichuan, Inner Mongolia, and Heilongjiang provinces (Figure 1). The infection and mortality rates in the different provinces are slightly different (Table 1) [6,7,11,13,14,15,16]. Further analysis of gout in geese in these different provinces showed that infection was frequent in geese <3 weeks old. Pathogenicity testing also confirmed that a novel GoAstV strain was highly pathogenic in goslings aged 1 to 15 days, but caused only mild symptoms in goslings aged 25 and 35 days [17]. Clinically, the highest positive rate in some provinces was 86.1% and the mortality rate exceeded 80%. In addition, the co-circulation of different types of GoAstV (GI and G2) may occur. Zhang et al. (2020) reported co-infection of both strains in the same animal, with some goose gout being the result of the synergistic action of two GoAstVs in the clinic [13]. Epidemiological investigation has shown that the detection frequency of G2 is slightly higher than that of G1. At present, there are two clinical reports of GoAstV infection in ducklings. The mortality rate reached 30% and both GoAstV strains belonged to G2 [9]. A challenge study demonstrated that GoAstV can cause visceral gout in chickens [18]. These studies have revealed the widespread dissemination of GoAstV in China and demonstrated the potential risk of transmission across species. However, further studies are needed to determine the host range and transmission route of GoAstV.

## 3. Genomic Characteristics

**Figure 2:**
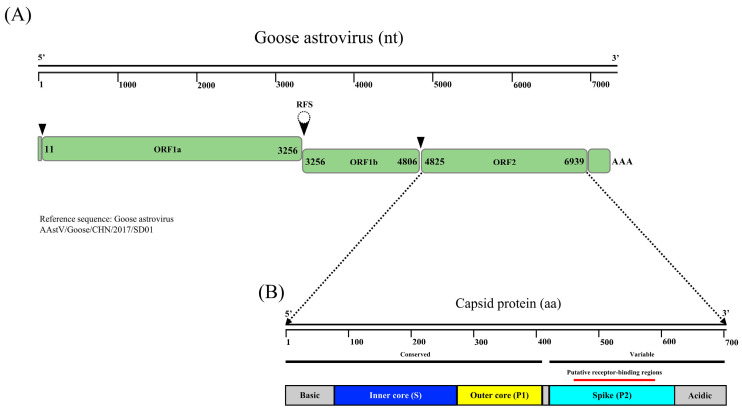
The genome structure of GoAstV. (**A**) The GoAstV genome contains three ORFs that encode three proteins (adapted from [24]). The sequences of ORF1a and ORF1b overlap. (**B**) Schematic of GoAstV capsid protein (CP) domain structure (adapted from [25]). The CP is colored by structural domains, with the inner core domain in blue, the outer core domain in yellow, and the spike domain in cyan.

GoAstVs are small non-enveloped viruses with a non-segmented, positive-sense, single-stranded RNA genome of 7.1 to 7.3 Kb. The genomic characteristics of GoAstV were analyzed with the AAstV/Goose/CHN/2017/SD01 strain as the reference strain [11]. The GoAstV genome is composed of a single-stranded sense RNA molecule of 7175 bp. The genome structure contains a 5′ and 3′ untranslated region (UTR), three open reading frames (ORFs; ORF1a, ORF1b, and ORF2), and a poly(A) tail (Figure 2A). ORF1a is approximately 3255 nucleotides (nt) in length and encodes a non-structural protein composed of 1085 amino acids (aa). The overlapping region between ORF1a and ORF1b contains the highly conserved “slippery heptamer” sequence 5′-AAAAAAC-3′ and a downstream hairpin structure. ORF1b is approximately 1551 nt in length and encodes an RNA-dependent RNA polymerase. The stop codon of ORF1b and the start codon of ORF2 are separated by a spacer sequence with a length of 18 nt. ORF2 is composed of 2115 nt that encodes a capsid protein. Capsid proteins contain a conserved N-terminal capsid core and a highly variable C-terminal spike domain (Figure 2B). The spike domain may be the target of host antibodies, including virus-neutralizing antibodies. A study of human astrovirus also showed that the spike domain contains the putative receptor domain of astrovirus [22]. In addition, the aa sequence of the ORF2 region is used as the basis for the classification of astroviruses. Astroviruses with aa sequence homology of the ORF2 protein > 75% are classified as the same species [23].

## 4. Phylogenetics and Evolution

The wide host range of astroviruses and the high degree of genetic diversity in the *Astroviridae* family have complicated attempts at a unified classification method. Astroviruses are classified within the *Astroviridae* family, which, initially, comprised a single genus (*Astrovirus*) based on virion morphology. In 2004, two genera were recognized: MAstV and AAstV. In 2011, the International Committee for Taxonomy of Viruses confirmed three Avastrovirus species: Avastrovirus 1 (AAstV-1), Avastrovirus 2 (AAstV-2), and Avastrovirus 3 (AAstV-3). The three species originally recognized within the genus were AAstV-1, comprising turkey astrovirus 1 (TAstV-1); AAstV-2, comprising avian nephritis virus 1 and 2; and AAstV-3, comprising turkey astrovirus 2 and duck astrovirus C-NGB [26]. The current classification scheme is based on phylogenetic analysis of the aa sequence of the full-length ORF2 region of the genome that encodes the capsid protein. However, the limited number of available capsid sequences makes consistent classification difficult, especially since some novel viruses have not yet been completely sequenced. There are many unclassified astroviruses, including turkey astrovirus type 3, chicken astrovirus (CAstV) A and B, duck astrovirus (DAstV) type 2 and 3, and some astroviruses isolated from wild birds.

**Figure 3:**
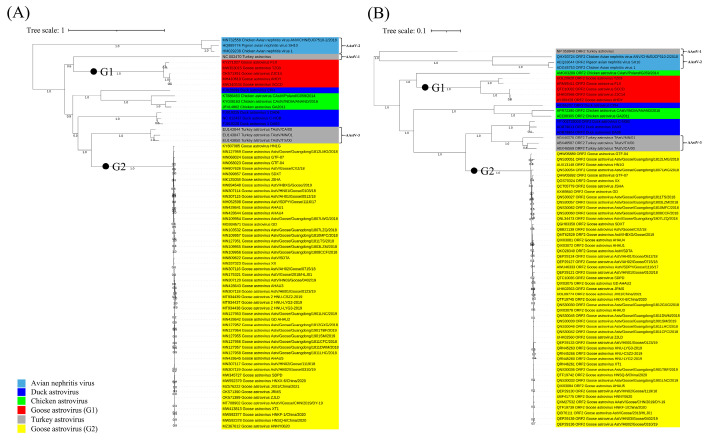
Phylogenetic analysis of GoAstV. (**A**) Phylogenetic analysis of GoAstV strains based on the whole genome. (**B**) Phylogenetic analysis of GoAstV strains based on the ORF2 aa sequence. Phylogenetic trees were generated based on whole genome and the ORF2 aa sequence using the neighbor-joining method with 1000 bootstrap replicates as determined with MEGA 6.0 software.

GoAstV has not yet been included in the official taxonomy [7,11]. In this review, 70 reference avian astroviruses are used for the phylogenetic analysis (Table S1). Phylogenetic trees of the full-length gene sequence and ORF2 aa sequence revealed that different avian astrovirus species were closely related, while GoAstV strains were classified as G1 and G2, which were distantly related. Phylogenetic analysis of the whole genome sequences revealed that G2 was closely related to AAstV-3, while G1 was closely related to AAstV-1 (Figure 3A). Phylogenetic analysis of the aa sequence in the ORF2 region showed that G2 was closely related to DAstV and AAstV-3, while G1 was closely related to CAstV (GenBank accession no. AMO03289) (Figure 3B). 

Astroviruses with aa sequence homology of the ORF2 protein >75% are classified as the same species. However, sequence alignment analysis revealed notable differences between G1 and G2, as the homologies of the full-length and ORF2 aa sequences between G1 and G2 were only 53.2% to 53.6% and 42.1% to 44.0%, respectively. Even if the sequence homology between the G1 and G2 groups is very low, recent reports revealed that gout-associated GoAstV strains were all clustered in G2, while the GoAstV strain TZ03 in group G1 can also replicate the symptoms of gout-associated GoAstV strains [12]. Further sequence alignment analysis may be important in understanding the same pathogenic characteristics of different types of astroviruses.

**Figure 4:**
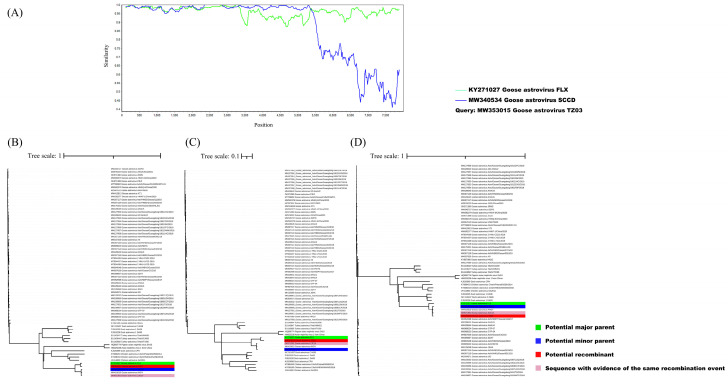
Identification of recombination events between the major parent strain, FLX strain (green), and the minor parent strain, SCCD strain (blue), which led to the recombinant TZ03 (red). (**A**) Simplot evidence for the recombination origin based on the pairwise distance, modeled with a window size of 200, step size of 20, and 100 bootstrap replicates. (**B**–**D**) A fast neighbor-joining (NJ) tree (1000 replicates, Kimura two-parameter distance) was constructed using the regions derived from the minor parent strain (1–3331 and 5445–8243) (**B**), the non-recombinant region (**C**), and recombination region (3330–5444) (**D**). Note. The potential recombination event was detected using RDP (*p* = 3.333 × 10^−23^), GENECONV (*p* = 1.179 × 10^−38^), BootScan (*p* = 2.284 × 10^−50^), MaxChi (*p* = 2.631 × 10^−12^), Chimaera (*p* = 4.845 × 10^−5^), SiSscan (*p* = 7.945 × 10^−53^), 3Seq (*p* = 4.913 × 10^−52^) methods.

The whole-genome sequence of the reference avian astroviruses from GenBank was used for the recombination analysis. Sequence alignment was performed using the ClustalX program and detection and analysis were carried out using the Recombination Detection Program [27], GENECONV [28], BOOTSCAN [29], MaxChi [30], CHIMAERA [31], and SISCAN [32] methods embedded in RDP4. The breakpoint positions of the recombination event are presented in Table S2. A recombination event that occurred between the SCCD strain and FLX strain led to the generation of the recombinant TZ03. The SimPlot of the recombination event is presented in Figure 4A, in which the FLX and SCCD strains were used as the major parent strain and minor parent strain, respectively. The recombination event was confirmed by the fast neighbor-joining trees that were constructed using the regions derived from the minor parent strain (5445–3331) (Figure 4B), the non-recombinant region (Figure 4C), and the recombinant region (3330–5444) (Figure 4D).

## 5. Pathogenicity of GoAstV

**Figure 5:**
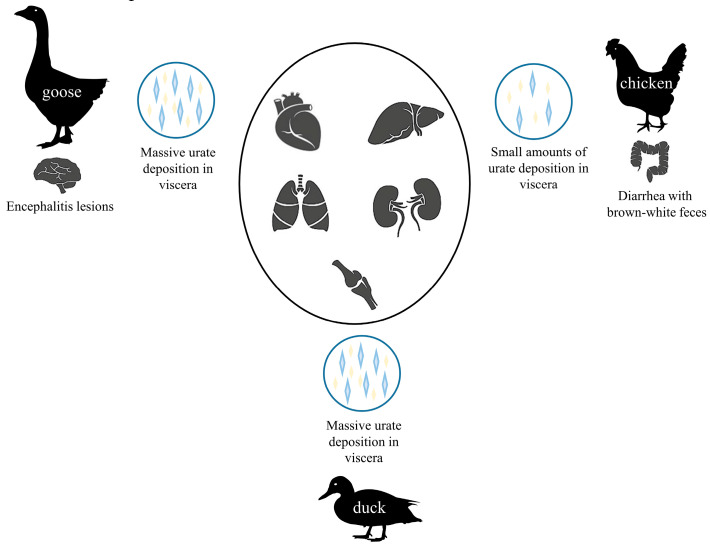
Pathogenicity of 3 different types of animal (goose, duck, chicken) infected with GoAstVs.

The reported mortality rates of GoAstV were 100% in inoculated goose embryos and 20%–50% in infected goslings aged 4 to 21 days [7,8,11,13,18,20]. GoAstV-infected goslings developed typical clinical symptoms of infection, such as slowed mobility and depression. As the infection progressed, affected goslings displayed signs of depression, loss of appetite, white feces, and lethargy and often died. At necropsy, obvious urate deposition was observed in the kidneys, joint cavities, and liver surface of goslings (Figure 5). Pathological damage was most severe to the kidneys, which were hemorrhagic and swollen. It is generally believed that GoAstV mostly targets the kidneys. Histopathological analysis revealed necrosis and abscission of renal tubular epithelial cells, inflammatory cell infiltration of the renal tissue, vacuolar degeneration of liver cells accompanied by inflammatory cell infiltration, disintegration and necrosis of spleen lymphocytes, microglia proliferation in the cerebral cortex, and dead nerve cells wrapped by microglia. The cecal villi were significantly shortened in goslings with gout, which inhibited the function of the intestinal mucosal barrier. In addition, encephalitis lesions were observed in the dead goslings [11,12,33]. The determination of viral titers in different organs showed that GoAstV infects a wide range of tissues in goslings, including the kidneys, liver, spleen, lung, brain, and heart. GoAstV infection is highly pathogenic to goslings aged 1 to 15 days, while those infected at 25 to 35 days only developed mild symptoms. GoAstV is shed in feces for up to 15 days, indicating that transmission in geese occurs through the fecal–oral route [17]. In addition, GoAstV infection may also be vertically transmitted [34].

Recent reports revealed that GoAstV could also infect ducklings and chickens. The clinical signs of infected ducks are similar to those of goslings and include depression, loss of appetite, white feces, and death at 4 to 5 days post-infection. Autopsy results showed that gross injury was also similar to that of infected goslings, including urate deposition in the joints and internal organs (heart, liver, kidneys, spleen, and lungs). Histological analysis demonstrated degeneration and necrosis in renal epithelial cells, glomerulonephritis, vacuolar degeneration of hepatocytes, and severe mucosal necrosis in the proventriculus [9,10].

A challenge study demonstrated that chickens infected with GoAstV developed the clinical symptoms of shedding brown-white feces and enlarged leg joints. At necropsy, the kidneys were swollen and the liver and ureter showed slight to mild urate deposition. Real-time polymerase chain reaction analysis confirmed that the chickens were successfully infected and GoAstV persisted in the body for at least 8 days [18]. The similar clinical symptoms of GoAstV infection in ducks, chickens, and goslings indicates the ability of the virus to cross the species barrier.

## 6. Pathogenesis of GoAstV

Carbamoyl phosphate synthetase and arginase are enzymes involved in the urea cycle. Poultry lack carbamoyl phosphate synthetase and arginase. Thus, ammonia is not converted to urea, but rather is used for synthesis of purines, xanthine, hypoxanthine, and finally uric acid. The latter is excreted in urine. Because uric acid is difficult to dissolve in water, it easily reacts with calcium and sodium to form calcium and sodium uric acid, which are deposited on the surface of the viscera, renal tubules, and articular cavities. Hence, poultry are more prone to hyperuricemia and gout [35]. 

**Figure 6:**
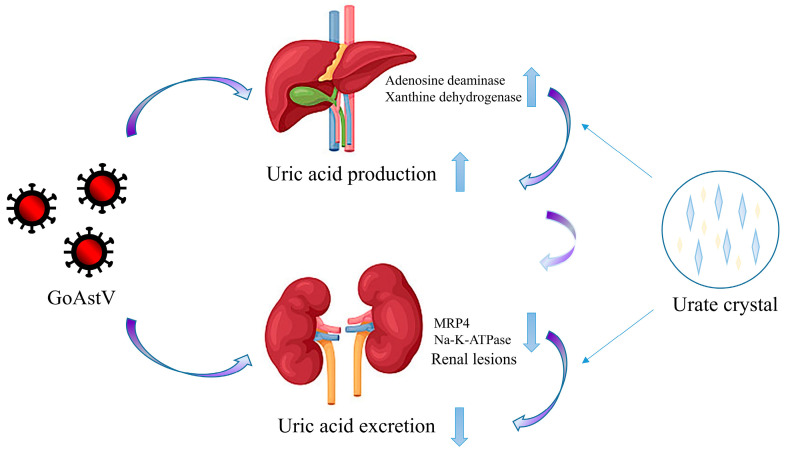
Pathogenesis of liver and kidney in gosling gout induced by GoAstV.

The pathogenesis of GoAstV-induced gout in goslings is due to excessive uric acid production, decreased excretion of uric acid, and renal injury (Figure 6). The pathogenicity of GoAstV infection in goslings includes significantly increased serum levels of uric acid at 14 days post-inoculation. A previous study found that the enzyme activities and expression of xanthine dehydrogenase (XOD) and adenosine deaminase (ADA) in the livers of GoAstV-infected goslings were significantly higher than in the same tissue of the control group. After GoAstV infection, the activity and expression of enzymes (XOD, ADA) related to uric acid production are increased in goslings, resulting in increased uric acid production, which may be important causes of hyperuricemia and gout in infected geese [36,37]. In addition, because uric acid is mostly excreted via the kidneys, organic anion transporter, multidrug-resistance-associated protein 4 (MRP4), sodium-dependent phosphate transport protein 1, and sodium–potassium pumps are the main transporters of uric acid in the renal excretion system of poultry [38,39]. A recent study showed that GoAstV infection significantly reduced mRNA expression of MRP4 and Na-K-ATPase activity, resulting in reduced excretion of uric acid by the kidneys [37]. In addition, GoAstV infection caused lesions to form on the kidneys and decreased renal excretion, which contributed to hyperuricemia and gout formation [11,33,40]. Taken together, the evidence indicates that GoAstV infection in goslings causes increased uric acid production by purine metabolism and decreased excretion of uric acid by the kidneys, resulting in the accumulation of uric acid, eventually leading to hyperuricemia and gout.

## 7. Detection Methods

Although time consuming and labor intensive, virus isolation and identification are the gold standard for the detection of many infectious diseases. GoAstV has been isolated from subcultures of goose embryos, chicken hepatocellular carcinoma (LMH) cells, chicken embryo fibroblast (DF-1) cells, and goose embryo kidney cells [10,11]. GoAstV was purified and observed by electron microscopy, which revealed a six-pointed star pattern of virions with a diameter of 28 to 30 nm [7]. Reverse transcription-polymerase chain reaction (RT-PCR) methods have been developed for rapid detection of GoAstV [20]. TaqMan-based quantitative RT-PCR has also been employed to detect and quantify GoAstV, as this method is highly sensitive and specific with a lower limit of detection for GoAstV of 33.4 copies/µL [19]. A reverse-transcription loop-mediated isothermal amplification method is developed for rapid detection of GoAstV by incubation at 60 °C for 60 min [21]. The sensitivity of this method for detection of GoAstV is 10-fold greater than that of conventional RT-PCR. In addition, metagenomic sequencing technology was also used as a powerful tool for detection of various pathogens, including GoAstV [12]. Among immunological methods, the enzyme-linked immunosorbent assay was established for the detection of serum antibodies against GoAstV [41].

## 8. Prospects of GoAstV

The prevalence of GoAstV in geese in recent years has led to a large-scale outbreak of gout in goslings, resulting in substantial economic losses. GoAstV research is still in the primary stage. Presently, strict biosafety measures are the main prevention and control strategies. Elucidation of the mechanism underlying GoAstV-induced gout in goslings, vaccine research, and the development of diagnostic and control technologies will be key directions in future GoAstV research. 

Considering relevant studies of other astroviruses, the spike region of capsid protein may be the target of virus-neutralizing antibody, which plays an important reference role in the design of the GoAstV subunit vaccine, compared with traditional vaccines, the preparation of safe, efficient, and rapid subunit vaccines may be the most timely response strategy to solve emerging viruses, including GoAstV. In addition, the spike region may have the receptor binding domain of astrovirus [23,42]. The receptor of astrovirus has not been determined and it is unclear whether different species of astrovirus use co-receptors. However, the similar viral structure of different species of astrovirus may provide a reference for the study of the GoAstV receptor. Further structural analyses of the spike region in GoAstV will be important for the identification of the GoAstV receptor binding region and the elucidation of the mechanism of invading cells.

GoAstV has a wide range of tissue tropism. In one study, encephalitis lesions were observed in deceased goslings inoculated with GoAstV, along with the detection of GoAstV RNA in the brain tissue [11]. Given the numerous reported cases of AstV-associated encephalitis and meningitis in humans and mammals [43,44], the results in goslings suggest that astrovirus infection of brain tissue may be a common feature of mammalian and avian astroviruses. Compared with human and other mammalian astroviruses, GoAstV is relatively easy to isolate and culture. Using GoAstV to reveal the pathogenic characteristics and mechanism of astroviruses’ damage to brain tissue may provide a reference for the study of brain tissue damage mechanism in mammalian astroviruses. Therefore, the neurologic infection of GoAstV is worthy of further investigation.

GoAstV has been transmitted in different species and similar pathogenic characteristics have been successfully replicated in ducks and chickens [9,18]. While GoAstV has caused serious economic losses in the breeding of geese and ducks, there are no reports of serious losses to chicken farms. In addition, both groups of GoAstV are more closely related phylogenetically to TAstV than to other groups, suggesting that the transmission of GoAstV may also be related to turkeys. Li et al. (2021) found that GoAstVs emerged very recently based on Bayesian inference analyses, around 2011, and the possible ancestral country of origin of this virus is China [18]. However, more sequences from additional regions may need to be analyzed to identify the exact origin of GoAstV. Understanding the origin of GoAstV would not only help to deepen the understanding of this new agent, but would also help prevent/limit the emergence of other similar zoonotic pathogens. Addressing the question about the origin of GoAstV and finding measures to limit the emergence of similar viruses (for example, limitation of multi-species breeding, reinforcement of sanitary measures, limitation of contacts between farm animals and wild animals, etc.) may be the key directions for GoAstV research, which is of great significance for containing the occurrence of similar cross-species transmission of viruses in the future. 

## Figures and Tables

**Table 1:** Summary of different epidemiology parameters from the different outbreaks of GoAstV between 2014 and 2019 in China.

Date	Province	Days Old	Positive Rate (%)	Morbidity (%)	Mortality Rate (%)	Representative Strain	Genotypes	Species	References
2014/06	Hunan	15	—	—	—	FLX	G1	goose	[8]
2016/01—2017/12	—	—	4.76	—	—	GsFJ2017	G2	goose	[19]
2017/02	Shandong	5-21	—	—	20–30	AAstV/Goose/CHN/2017/SD01	G2	goose	[11]
2017/03—2017/12	—	4-21	81.50	—	—	AstV/SDPY/Goose/1116/17	G2	goose	[6]
2017/10—2017/12	Anhui	—	72.90	—	—	AHAU1	G2	goose	[20]
2017-2018	Shandong	—	55.56	—	—	SD18	G2	goose	[14]
2017/10—2019/5	Guangdong	5-12	—	—	35-40	SDPD+SCCD	G1+G2	goose	[13]
	Shandong	5-50	—	—	1-40		G1+G2	goose	
	Sichuan	8-15	—	—	20-40		G1+G2	goose	
	Zhejiang	10	—	—	30-40		G2	goose	
	Anhui	7-50	—	—	2-50		G1+G2	goose	
	Jiangsu	5-35	—	—	10-50		G1+G2	goose	
	Hubei	15	—	—	35-40		G1+G2	goose	
	Liaoning	10-20	—	—	40-50		G1+G2	goose	
	Inner Mongolia	10	—	—	30-40		G1+G2	goose	
	Hebei	7-15	—	—	45-50		G1+G2	goose	
	Henan	5-20	—	—	35-40		G1+G2	goose	
2018	Anhui	—	39.50	—	—	AH/2018	G2	goose	[21]
2019/05	Hunan	5-8	—	10-20	—	HNU-LYG3-2019	G2	goose	[18]
2019/08	Fujian	5-20	—	40	80	FJ-NP	G2	goose	[15]
2019/08	Jiangsu	7-21	—	40	—	TZ03	G1	goose	[12]
2019/03	Shandong	2-8	—	—	30	SDXT	G2	duck	[9]
2019/07—2019/10	Shandong, Jiangsu, Henan	—	86.10	—	—	AstV-SDTA	G2	duck	[16]

## Data Availability

Data sharing not applicable.

## Supplementary Materials

The following supporting information can be downloaded at: https://www.mdpi.com/article/10.3390/v14081759/s1, Table S1: The reference AstV strains used in this study; Table S2. The breakpoint positions of recombination events in our study.
