# Supplementary material for: Goose Astrovirus in China: A Comprehensive Review
Source: Viruses. 2022 Aug 12;14(8):1759. doi: 10.3390/v14081759 (PMC9416409; doi:10.3390/v14081759)
Supplement: Supplementary file 1 [file viruses-14-01759-s001.zip › Table S1.pdf]

Table S1. The reference AstV strains used in this study.

Strains name	Hosts	GenBank accession no.	Collection date	Countries
TAstV/TX/00	Turkey	EU143850	-	USA
-	Turkey	NC_002470	-	USA
TAstV/CA/00	Turkey	EU143844	-	USA
ANV/CHN/BJCP510-2/2018	Chicken	MN732558	2018	China
DA93	Duck	FJ919228	-	China
C-NGB	Duck	NC_012437	2008	USA
DA06	Duck	FJ919225	-	China
CPH	Duck	KJ020899	2013	China
CAstV/Poland/G059/2014	Chicken	KT886453	2014	Poland
GA2011	Chicken	JF414802	2011	USA
CAstV/INDIA/ANAND/2016	Chicken	KY038163	2016	India
1	Avian_nephritis	HM029238	2009	China
SH10	Pigeon_avian_nephritis	HQ889774	2010	China
TAstV/MN/01	Turkey	EU143847	-	USA
ZJLD	Goose	OK571389	2020	China
TZ03	Goose	MW353015	2019	China
HNNY0620	Goose	MZ367612	2020	China
HNXX-6/China/2020	Goose	MW592379	2020	China
HNSQ-6/China/2020	Goose	MW592378	2020	China
HNKF-1/China/2020	Goose	MW592377	2020	China
SDPD	Goose	MW345727	2019	China
SCCD	Goose	MW340534	2019	China
AstV/SDTA	Goose	MN809622	-	China
SDXT	Goose	MN399857	-	China
AstV/SDPY/Goose/1116/17	Goose	MH052598	2017	China
ZJC14	Goose	OK571391	2020	China
JRMS	Goose	OK571390	2020	China
JX01/China/2021	Goose	MZ576222	2021	China
AAstV/Goose/CHN/2019/DY-19	Goose	MT708902	2019	China
XT1	Goose	MW413813	2020	China
Astv/Goose/Guangdong/1810MFC/2018	Goose	MN109957	2018	China
Astv/Goose/Guangdong/1808CCF/2018	Goose	MN109956	2018	China
Astv/Goose/Guangdong/1803LZM/2018	Goose	MN109955	2018	China

Astv/Goose/Guangdong/1807LWG/2018	Goose	MN109954	2018	China
Astv/Goose/Guangdong/1812LMG/2018	Goose	MN127959	2018	China
Astv/Goose/Guangdong/1811LHC/2018	Goose	MN127958	2018	China
Astv/Goose/Guangdong/1811DWM/2018	Goose	MN127957	2018	China
Astv/Goose/Guangdong/1811CFC/2018	Goose	MN127956	2018	China
Astv/Goose/Guangdong/1901SM/2019	Goose	MN127955	2019	China
Astv/Goose/Guangdong/1901TBF/2019	Goose	MN127954	2019	China
Astv/Goose/Guangdong/1901LNC/2019	Goose	MN127953	2019	China
Astv/Goose/Guangdong/1812GXG/2018	Goose	MN127952	2018	China
Astv/Goose/Guangdong/1811TS/2018	Goose	MN127951	2018	China
Astv/Goose/Guangdong/1807LZQ/2018	Goose	MN103532	2018	China
AstV/HB02/Goose/0310/19	Goose	MN307119	2019	China
AstV/HB01/Goose/0123/19	Goose	MN307118	2019	China
AstV/HN02/Goose/1119/18	Goose	MN307117	2018	China
AstV/AH02/Goose/0715/18	Goose	MN307116	2018	China
AstV/AH01/Goose/0512/18	Goose	MN307115	2018	China
AstV/HN01/Goose/0103/18	Goose	MN307114	2018	China
AstV/HBXG/Goose/2019	Goose	MN894548	2019	China
AHAU5	Goose	MN428645	2018	China
AHAU4	Goose	MN428644	2018	China
AHAU3	Goose	MN428643	2018	China
GD_AHAU2	Goose	MN428642	2018	China
AHAU1	Goose	MN428641	2018	China
AstV/Goose/2018/HLJ01	Goose	MN175321	2018	China
GTF-07	Goose	MN068024	2018	China

GTF-04	Goose	MN068023	2018	China
XX	Goose	MN337323	2018	China
JSHA	Goose	MK125058	2016	China
AstV/Goose/CXZ/18	Goose	MH807626	2018	China
HN1G	Goose	KY807085	2014	China
AHDY	Goose	MH410610	2014	China
FLX	Goose	KY271027	2014	China
GD	Goose	MG934571	2017	China
FLX	Goose	KY271027	2014	China
HNU-CSZ2-2019	Goose	MT934439	2019	China
HNU-LYG3-2019	Goose	MT934438	2019	China
HNU-LYG2-2019	Goose	MT934437	2019	China
AAstV/Goose/CHN/2017/SD01	Goose	MF772821	2017	China
