# Supplementary material for: Goose Astrovirus in China: A Comprehensive Review
Source: Viruses. 2022 Aug 12;14(8):1759. doi: 10.3390/v14081759 (PMC9416409; doi:10.3390/v14081759)
Supplement: Supplementary file 1 [file viruses-14-01759-s001.zip › Table S2.pdf]

Table S2. The breakpoint positions of recombination events in our study.

Breakpoint Positions in Recombinant Sequence					Detection Methods								
Begin	End	Recombinant Sequence	Minor Parental Sequence	Major Parental Sequence	RDP	GENECONV	Bootscan	Maxchi	Chimaera	SiSscan	PhylPro	LARD	3Seq
3330	5444	TZ03	SCCD	FLX	3.333E -23	1.179E-38	2.284 E-50	2.631E-12	4.845E-05	7.945E-53	NS	NS	4.913E-52

Note. Minor Parent = Parent contributing the smaller fraction of sequence. Major Parent = Parent contributing the larger fraction of sequence. NS = No significant *P*-value was recorded for this recombination event using this method.
